# PlaToLoCo: the first web meta-server for visualization and annotation of low complexity regions in proteins

**DOI:** 10.1093/nar/gkaa339

**Published:** 2020-05-18

**Authors:** Patryk Jarnot, Joanna Ziemska-Legiecka, Laszlo Dobson, Matthew Merski, Pablo Mier, Miguel A Andrade-Navarro, John M Hancock, Zsuzsanna Dosztányi, Lisanna Paladin, Marco Necci, Damiano Piovesan, Silvio C E Tosatto, Vasilis J Promponas, Marcin Grynberg, Aleksandra Gruca

**Affiliations:** Department of Computer Networks and Systems, Silesian University of Technology, Akademicka 16, 44-100 Gliwice, Poland; Institute of Biochemistry and Biophysics PAS, Pawinskiego 5A, 02-106 Warsaw, Poland; Faculty of Information Technology and Bionics, Pázmány Péter Catholic University, Práter u. 50/A, 1083 Budapest, Hungary; Research Centre for Natural Sciences, Magyar Tudósok Körútja 2, 1117 Budapest, Hungary; Structural Biology Group, Biological and Chemical Research Centre, Department of Chemistry, University of Warsaw, Żwirki i Wigury 101, 02-089 Warsaw, Poland; Faculty of Biology, Johannes Gutenberg University Mainz, Hans-Dieter-Hüsch-Weg 15, 55128 Mainz, Germany; Faculty of Biology, Johannes Gutenberg University Mainz, Hans-Dieter-Hüsch-Weg 15, 55128 Mainz, Germany; ELIXIR, Wellcome Genome Campus, Hinxton, Cambridgeshire CB10 1SD, UK; Department of Biochemistry, ELTE Eötvös LorándUniversity, Budapest, Pázmány Péter stny 1/c 1117, Budapest, Hungary; Department of Biomedical Sciences, University of Padova, Via Ugo Bassi 58/B, 35131 Padova, Italy; Department of Biomedical Sciences, University of Padova, Via Ugo Bassi 58/B, 35131 Padova, Italy; Department of Biomedical Sciences, University of Padova, Via Ugo Bassi 58/B, 35131 Padova, Italy; Department of Biomedical Sciences, University of Padova, Via Ugo Bassi 58/B, 35131 Padova, Italy; Bioinformatics Research Laboratory, Department of Biological Sciences, University of Cyprus, P.O. Box 20537, Nicosia, CY 1678, Cyprus; Institute of Biochemistry and Biophysics PAS, Pawinskiego 5A, 02-106 Warsaw, Poland; Department of Computer Networks and Systems, Silesian University of Technology, Akademicka 16, 44-100 Gliwice, Poland

## Abstract

Low complexity regions (LCRs) in protein sequences are characterized by a less diverse amino acid composition compared to typically observed sequence diversity. Recent studies have shown that LCRs may co-occur with intrinsically disordered regions, are highly conserved in many organisms, and often play important roles in protein functions and in diseases. In previous decades, several methods have been developed to identify regions with LCRs or amino acid bias, but most of them as stand-alone applications and currently there is no web-based tool which allows users to explore LCRs in protein sequences with additional functional annotations. We aim to fill this gap by providing PlaToLoCo - PLAtform of TOols for LOw COmplexity—a meta-server that integrates and collects the output of five different state-of-the-art tools for discovering LCRs and provides functional annotations such as domain detection, transmembrane segment prediction, and calculation of amino acid frequencies. In addition, the union or intersection of the results of the search on a query sequence can be obtained. By developing the PlaToLoCo meta-server, we provide the community with a fast and easily accessible tool for the analysis of LCRs with additional information included to aid the interpretation of the results. The PlaToLoCo platform is available at: http://platoloco.aei.polsl.pl/.

## INTRODUCTION

Low complexity regions (LCRs) are stretches of protein sequences that are characterized by a less diverse amino acid composition compared to the typical sequence diversity observed in proteins. LCRs can consist of single amino acid repeats (so-called homorepeats, homopolymeric regions), repetitive regions which consist of patterns of residues that are adjacent to each other (such as direpeats, tandem repeats or imperfect repeats), or compositionally biased regions (regions that lack a particular pattern but are enriched in a few amino acid types) (1).

For many years, LCRs were ignored by the scientific community, treated as a non-functional part of a proteome (or ‘junk’) (2). However, recent research shows that low complexity sequences may co-occur with intrinsically disordered regions and these represent a significant portion of proteins that lack 3D structural information, the so-called dark proteome (3). Recent studies have also shown that LCRs are highly conserved in many organisms (4). While the function of most LCRs is still a mystery, recent evidences suggest that LCRs often play important roles in structure stability preservation (5), adhesion (6), transduction of conformational information (7), membrane interactions (8), DNA binding (9), the binding of metals by cysteine, histidine, or charge clusters (10), and in driving the formation of membraneless organelles through phase separation (11). LCRs are also directly involved in the development of various diseases, including neurodegenerative diseases and cancer (12,13).

Here we present PlaToLoCo - Platform of Tools for Low Complexity, which is a meta-server that integrates and collects the output of five different state-of-the-art tools for the discovery of LCRs. These methods (SEG, CAST, fLPS, SIMPLE and GBSC) were selected because they represent a range of approaches to detect low complexity regions. SEG (14) is the most commonly used tool for masking low complexity regions and it is often used by the similarity search tool BLAST (15). CAST (16) and fLPS (17) are methods focused on searching for compositionally biased regions. SIMPLE (18) is designed to detect highly cryptic (non-tandem) repeats and GBSC (manuscript in preparation) is a graph-based method also designed for finding repeats in protein sequences, including non-perfect repeats. PlaToLoCo not only searches for low complexity regions but also for the first time provides functional annotations for these regions such as domain detection, transmembrane segment prediction, and calculation of amino acid frequencies. In addition, the consensus or intersection of the results of the search on the query sequence can be obtained by the user.

To our best knowledge there currently exists only one tool with a similar functionality which is LCR-eXXXplorer (19). This tool offers precalculated results of CAST and SEG (with their default settings) for all UniProtKB/SwissProt database (20) entries (release 2015_01), while features currently annotated in UniProtKB for these entries are retrieved and displayed along with the detected LCRs. The main disadvantage of this approach is the limited number of LCR detection methods and the fact that it does not allow users to submit their own protein sequences.

## MATERIALS AND METHODS

Different definitions of LCRs have been proposed in the literature leading to various tools for their detection (1). PlaToLoCo incorporates widely used LCR detection methods, which are representatives of the main formulations that have been proposed for this task. In this section, we present a short description of the methods implemented in the PlaToLoCo server.

*SEG* has been the *de facto* standard for LCR detection for at least two decades. Its popularity stems from multiple factors, including (a) a simple—yet rigorous – mathematical formulation, (b) availability of the source code and (c) its inclusion as a default masking option within the NCBI-BLAST package. Briefly, SEG uses a two-pass, sliding-window approach for calculating a measure of information content (sequence complexity or Shannon entropy) along a sequence in overlapping peptides of fixed length (trigger window length – *W*). SEG initially detects candidate windows satisfying a strict predefined threshold (trigger complexity – *K*_2_(1)). Overlapping candidates are merged and extensions are made during the second pass, ending with ‘contigs’ of complexity lower than a more relaxed threshold (extension complexity – *K*_2_(2) > *K*_2_(1)). A final optimization step performs a brute-force search within each contig for the subsequence with the minimum occurrence probability. The number and sequence properties of the detected LCRs in a dataset (e.g. LCR length distribution) clearly depend on the choice of *W*, *K*_2_(1) and *K*_2_(2), with the authors proposing specific parameter settings for particular purposes (14).

*CAST* detects compositionally biased regions in a query sequence in an implicit manner by identifying regions that exhibit high similarity to homopolymers of any type of proteinogenic amino acid. Local sequence similarities against homopolymers (signifying LCR candidates) are detected using a space- and time-efficient implementation of the Smith-Waterman algorithm (21) using the BLOSUM62 scoring matrix and an infinite gap penalty. A detection step is followed by masking of the region of highest similarity; this procedure is then iterated until the highest detected similarity score values fall below a predetermined threshold.

*SIMPLE* identifies short motifs in a sliding window with frequencies that are higher than those in sliding windows of the same size in randomized versions of the sequence under study. Repeated motifs are given a score and those that score higher than randomized sequences are marked as significant. Additionally, an average score for all the detected windows in a sequence is generated as an indicator of how repetitive that sequence is as a whole. The method was originally published in 1986 for DNA sequences (22) and subsequently updated (23), and extended for usage with protein sequences (18).

*fLPS* (fast Lowest Probability Subsequences) detects compositional biases (CBs) in protein sequences. Such regions were found in functionally important proteins, e.g. in prion-like proteins (24). The underlying algorithm is based on the LPS algorithm (25), although fLPS is significantly more computationally efficient. This method has three main parts. First, a high bias probability threshold *t* is used to identify segments with skewed amino acid composition (QUICKSCAN). Overlapping regions are merged into contigs. Then, contigs are scanned for the LPSs using windows sizes from maximum *M* to minimum *m* (MINIMIZATION). Finally, LPSs of different residues are combined if their *P*-values will be higher than the combined LPS *P*-value (MERGE). These segments are trimmed or extended if their *P*-values can be further decreased.

*GBSC* (*Jarnot, P., Ziemska-Legiecka, J., Grynberg, M. and Gruca, A., in preparation*) identifies repetitive regions composed of one amino acid (homorepeats) or a few amino acids (STRs – short tandem repeats). This method is based on weighted paths of graphs built from consecutive 2-mers in the sequence. This algorithm also identifies imperfect homo/tandem repeats from protein sequences by scanning all the provided sequences. Because this method can detect imperfect repeats, insertions in between the repeats and mutations of amino acids within repetitive regions are less confounding to the method. The user can set the window size used to scan the sequences and the minimum number of occurrence of the repetitive pattern as parameters. The positions of tandem repeats and the information about the type of repeat are returned as outputs. The detailed description of the GBSC method is provided in the Supplementary material 1 (Suppl1).

## WEB SERVER DESCRIPTION

The PlaToLoCo web server available at http://platoloco.aei.polsl.pl takes a list of UniProt accession numbers or a list of protein sequences in FASTA format and the parameters of implemented methods. By default, all method parameters are set as suggested by the authors of the original papers, however, the web interface allows users to modify these values. Additionally, for SEG and fPLS, we also provide suggested, predefined parameter settings that narrow search results and are tailored to specific needs. For SEG the additional parameter settings are SEG *intermediate* (*W* = 15, *K*_1_ = 1.9 and *K*_2_ = 2.5) and SEG *strict* (*W* = 15, *K*_1_ = 1.5 and *K*_2_ = 2.8). According to previous reports, SEG *intermediate* is optimized for detecting longer and more repetitive low complexity regions in eukaryotes (26), while SEG *strict* ensures that the regions identified correspond to strongly compositionally biased sequences while also allowing for substantial sequence diversity (27). For fLPS, we provide the additional parameter set fLPS *strict* (*m* = 5, *M* = 25, and *t* = 0.00001) suggested by the method author as more suitable for detecting compositionally biased regions (17).

As the computation time may vary from a few seconds up to several hours depending on the number and length of submitted sequences and the load of the PlaToLoCo server, users are provided with jobIDs immediately after submitting the list of sequences. This unique identifier can be used to retrieve the results on demand at a later time as the results are stored on the server for seven days. When the submitted job is finished, a new panel is produced by the web server showing the summary of results, including LCR prediction results for all the submitted sequences.

**Figure 1.**
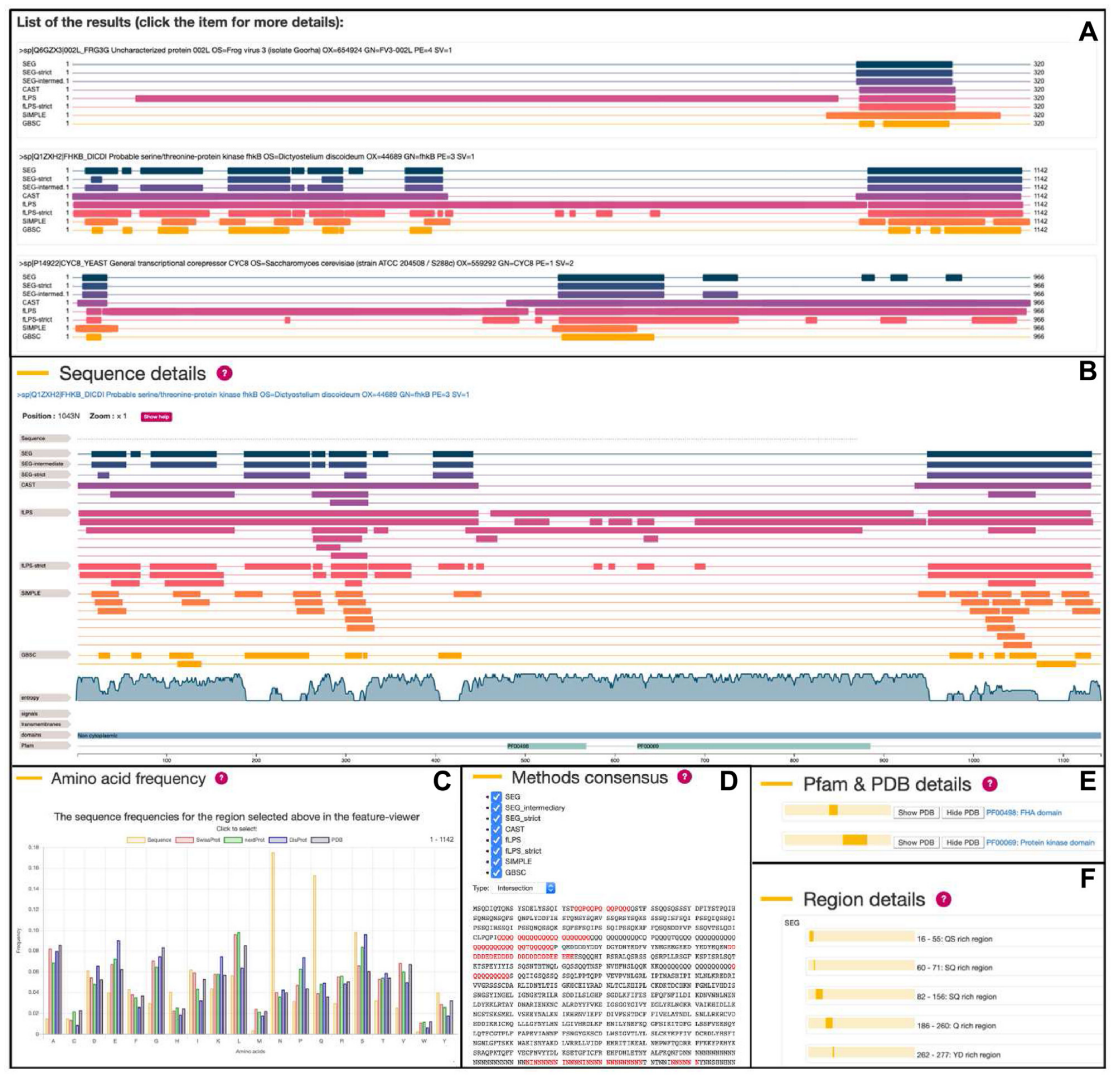
Layout of the results tab of the PlaToLoCo webserver. (**A**) summary tab, (**B**) sequence details, (**C**) amino acid frequency, (**D**) methods consensus, (**E**) Pfam and PDB details and (**F**) region details.

Further results and statistics are available in the *sequence details* panel (Figure 1A) which is activated by clicking on the summary of a particular query sequence. The panel offers a graphical representation of the LCRs detected by the selected methods (Figure 1B). The graph consists of three parts. The amino acid sequence is shown on top (while the sequence is zoomed). Below that the LCR sequences found by the different methods are presented, followed by the Shannon entropy computed over a sliding widow of size 7, and then the selected enrichment information. The positions in the query sequences are shown at the bottom of the graph. Below there is the *amino acid frequency* section which shows the amino acid distribution of the sequence compared to the amino acid frequencies in various databases such as Uniprot/SwissProt, nextProt (28), DisProt (29) and PDB (30) (Figure 1C). Users can personalize their results in the *methods consensus* panel, by selecting their preferred LCR detection methods and selecting either the consensus result using a strict definition calculated from the intersection of the selected results, or using a more permissive definition calculated from the union of the results (Figure 1D). The selected consensus results can also be downloaded by the user in fasta format. The next section is called *Pfam and PDB details* and it provides detailed information about the Pfam domains annotated to the analyzed sequence as well as PDB structures associated with that domain (Figure 1E). Finally, the *region details* panel shows the summary of all the predicted LCRs displaying the specific amino acids enriched in the queried sequences (Figure 1F).

### Implementation

In order to run the LCR identification methods we decided to unify the input and output of each method to simplify the design of the server under the wrapper module. An additional advantage of this approach is that the user can run all of the methods from the command line using unified fasta format sequences as input and receiving regions of interest as results in the same format. Other functionalities incorporated in PlaToLoCo for enriching results are: (a) Phobius (31), for simultaneously predicting secretory signal peptides and transmembrane helices, (b) PFAM (32) for displaying functional domains, and (c) amino acid frequencies in the query sequence compared to the UniProtKB/Swiss-Prot database frequencies.

PlaToLoCo uses several client-side Javascript libraries and components. The platform is based on the Angular.js library which enabled the creation of a responsive and intuitive application. It also utilizes: xml-js to parse data collected from the Pfam database, chart.js to present the amino acid frequencies chart shown in Figure 1C and feature-viewer (33) to present the sequence details shown in Figure 1B. The client communicates with the server using a RESTful API.

Server-side software was developed based on the FLASK library, which is a complete implementation of the RESTful service. Data is stored in content addressable storage (CAS). Session token and URL addresses for the results page are generated based on the query requests. We use SHA-1 to calculate the token from the requested data. This approach has several advantages. It is easy to implement and use and it allows the server to reuse previously calculated queries. The most frequent occurrence of this mechanism is the situation when the user starts an analysis and then closes the web page without copying the link/token for their job. As the user session ID is based on a token calculated from the query, it may be used to find this job if the same user submits the same query again.

The architecture design lets the user use the RESTful API directly, allowing programmable access to the PlaToLoCo server without using the convenient but restrictive web interface. Example commands in Python are also available on the webserver’s API page.

## CASE STUDY

PlaToLoCo is the first platform that enables the user to retrieve and examine multiple annotations of low complexity sequences at the same time. Different predictors featured in PlaToLoCo provide diversified perspectives on the problem of identification of low complexity regions. Having these different perspectives together allows a diverse coverage of cases of low complexity. This concept is illustrated here through several examples.

**Figure 2.**
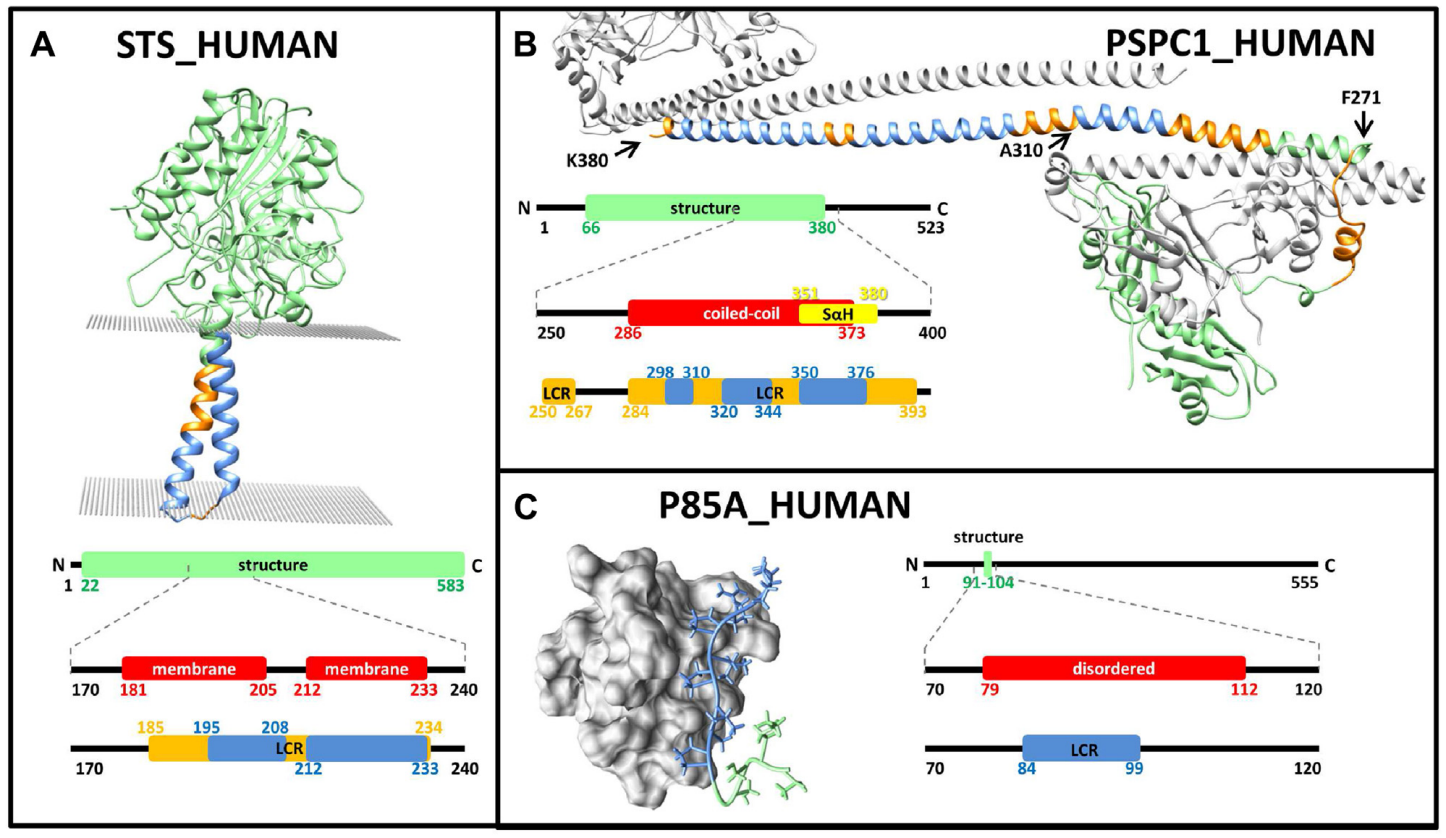
(**A**) The structure of steryl-sulfatase (PDB: 1p49). Membrane regions were predicted by CCTOP (red) (34). (**B**) The modelled structure of paraspeckle component multimers. Coiled-coil regions were predicted by DeepCoil (red) (35), Single-helices were predicted by CSAHdetec (yellow) (36). (**C**) Structure of SH3 domain and the p85 subunit of PI3-kinase. Disordered regions were predicted with IUPRED (red) (37). Blue: overlap of SEG, CAST and fLPS. Orange: overlap of CAST and fLPS – also highlighted on the structures.

Although LCRs are most commonly associated with intrinsically disordered regions (IDRs), there are a handful of exceptions that form stable structures. One exception is the set of α-helical transmembrane proteins that cross the lipid bilayer one or more times, which is unfavorable for (partially) charged functional groups, resulting in a skewed amino acid distribution. For example, steryl-sulfatase (STS_HUMAN, UniProt AC P08842) has two transmembrane regions (38,39), both detected by SEG, CAST and fLPS. Although these methods are unable to precisely detect region boundaries, it is clear that LCRs and IDRs are neither identical nor interchangable (Figure 2A).

Another example is PSPC1 (PSPC1_HUMAN, UniProt AC Q8WXF1), which multimerizes in multiple steps: the middle region of the protein forms a dimer with NONO or SFPQ via an unusual right-handed coiled-coil domain with hendecad motifs, elongated with a single α-helix. The second step has not been completely characterized, although a transition from a single α-helix to a weak coiled-coil with other PSPC1/NONO dimers has been proposed (40). Both the coiled-coils and the single α-helices have unusual residue distributions and the main forces driving the coiled-coil interactions are hydrophobic contacts supported via electrostatic interactions which results in a repeat pattern of 7 or 11 residues (41), while the single α-helices also rely on repeated charged residues that form salt bridges (42). These regions are predicted (35,36) to overlap with the LCRs and this has been confirmed by X-ray studies (43,44), and theoretical modelling (40) of the multimer (Figure 2B).

Another example of low complexity is a region of the PI3-kinase p85 subunit (P85A_HUMAN, UniProt AC P27986) that binds to an SH3 domain forming a stable structure (45). This peptide contains a significant number of prolines, likely to decrease the loss of entropy during binding. The interacting region is predicted to be an IDR (37) and is identified by several LCR tools. Moreover, the consensus of the SEG, CAST and fLPS results seems to identify the proline-rich interacting segment precisely (Figure 2C).

The inhibitory domain of the mineralocorticoid receptor is another fine example of how low complexity regions play a role in preserving structure (46). Likewise, Huntingtin (UniProt AC P42858) contains a poly-glutamine (polyQ) homorepeat that causes the disease phenotype when there are more than 36 glutamines present. Huntingtin has a stretch of glutamines from residue 18 to 38 and all predictors in PlaToLoCo correctly identify a reduction in sequence complexity around this region. Although, different predictors find other and different LCRs along the sequence of huntingtin.

Another protein, yeast RNA polymerase II subunit RPB1 (UniProt AC P04050) is essential to the formation of the RNA Polymerase II complex. This protein contains a sequence repeat in its C-terminus (residues 1549–1716). All methods in PlaToLoCo except SIMPLE identify this region as an LCR. GBSC in particular detects many subregions (different repeats), providing a higher level of granularity. This, together with the prediction from the Pfam database (reported in the Feature Viewer), helps in identifying the type of low complexity and provides additional information to other methods predictions.

Med1 (UniProt AC Q15648) is a component of the mediator complex and is involved in the transcriptional regulation of proteins that depend on RNA polymerase II. This protein is highly compositionally biased and contains intrinsically disordered regions from around residue 750 to its C-terminus. PlaToLoCo identifies LCRs from residue 550 onward, with CAST giving the whole region down to its C-terminus as a serine-rich region, while other predictors find more specific and sparse subregions of LC. In the feature viewer, the user gets an immediate and intuitive view of the differences between the methods’ predictions.

RNA binding protein FUS (UniProt AC P35637) is a mediator of many processes, namely RNA, transport, splicing and DNA repair. It is also involved in several diseases including amyotrophic lateral sclerosis (ALS) (47,48). FUS has long low complexity IDRs and can form liquid droplets (49–51) via liquid-liquid phase separation. PlaToLoCo predictors almost unequivocally agree that this protein is almost completely composed of low complexity regions, with the exception of a central region (from residue 260 to 370). Predictors identify the N-terminal low complexity domain of FUS, a highly conserved prion-like domain composed primarily of serine, tyrosine, glycine and glutamine rich regions.

The probable serine/threonine-protein kinase fhkB is a reviewed protein in UniProt (Q1ZXH2) from *Dictyostelium discoideum*. Two regions are annotated as domains and the rest of the protein is either considered a coiled coil or compositionally biased. PlaToLoCo gives greater insight into these regions. The differences between predictors are evidenced in Figure 1B. fLPS finds low complexity regions in the middle of the sequence while the other methods focus their prediction on the N- and C-termini, more or less where the domains from UniProt are annotated. CAST identifies two continuous regions at the termini, while the other predictors give a more fragmented and detailed prediction. Since low complexity does not have a unique definition, having multiple takes on the problem allows the user to explore the phenomena at the desired level of detail.

**Figure 3.**
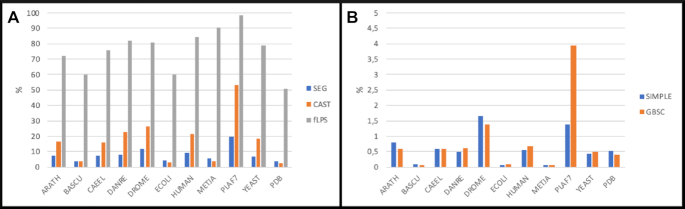
Panel **A**: proportion of detected low complexity and compositionally biased regions in various proteomes and in the PDB (detected LCRs to total number of residues) for SEG, CAST and fLPS. Panel **B**: proportion of repeat regions in various proteomes and in the PDB (detected repeats to total number of residues) for SIMPLE and GBSC. The exact number of residues found by each method is provided in the Supplementary material 2 (Suppl2) in Supplementary Table S2.

To have an overall picture of the distribution of LCRs, we ran PlaToLoCo on different proteomes (obtained from UniProt, March 2019, with cd-hit (52) 40% redundancy control) and used the PDB (non-redundant PDB chain set, March 2019, *P*-value cutoff of 10e−7) as a control. In general, LCRs and CBRs were more commonly detected compared to repeat regions (Figure 3). However different proteomes tended to have different varieties of LCRs. For example, fLPS identified roughly the same proportion of CB segments in all the genomes, while CAST predicted an unusually high proportion of CB segments in the *Plasmodium falciparum* genome. Interestingly, this parasite also seemed to have a higher frequency of repeats that were detected by GBSC and SIMPLE as compared to the other test genomes. We also analyzed several other performance measures such as method run time, the total number of residues found by each method, and the detected overlap between methods. These statistics are provided in the Supplementary material 2 (Suppl2).

## DISCUSSION

For many years low complexity regions were thought to be *junk* part of proteome. This resulted in a reduced development of tools and methods for their analysis. Most of the algorithms that have been developed to identify regions with LCRs or amino acid biases were developed in previous decades, and most of them are available only as standalone applications. Usually they are difficult or sometimes even impossible to install as they require outdated package dependencies. Furthermore, they typically provide only a list of discovered regions without any functional annotation. By developing the PlaToLoCo meta-server, for the first time, we provide the community with a fast and easily accessible tool for the analysis of LCRs, enriching them with additional information to aid the interpretation of the results.

In the future we plan to improve PlaToLoCo by providing additional functional annotations such as annotated protein regions from UniProt or predictions of protein disorder. We also plan to run PlaToLoCo against full proteomes and complete protein databases as having precomputed results for all UniProtKB proteins (or initially at least SwissProt entries) would reduce the execution time dramatically when submitting a UniProt AC as input.

Examining the overlap between the results of selected methods we notice that the output results may vary significantly when different methods are compared. This supports the conclusion that the PlaToLoCo platform provides a selection of methods that are designed to detect different types of LCRs. Since there is no single, universally accepted definition of LCRs and various methods attempt to analyse the statistical properties of the sequences or specific sequence patterns, it is important to provide the scientific community with a tool that allows the analysis of low complexity regions from different perspectives and on different granularity levels, covering a wide range of approaches to detect these regions.

## Supplementary Material

gkaa339_Supplemental_Files
