# Supplementary material for: PlaToLoCo: the first web meta-server for visualization and annotation of low complexity regions in proteins
Source: Nucleic Acids Res. 2020 May 18;48(W1):W77–84. doi: 10.1093/nar/gkaa339 (PMC7319588; doi:10.1093/nar/gkaa339)
Supplement: gkaa339_Supplemental_Files [file gkaa339_supplemental_files.zip › Suppl1.pdf]

SUPPLEMENTARY MATERIAL 1

GBSC - **G**raph **B**ased on **S**equence **C**lustering is a new method (*Jarnot P., Ziemska-Legiecka J., Grynberg M., Gruca A, in preparation*) that can be used to identify low complexity regions of protein sequences. In particular, it is designed to find strings of imperfect and/or fused repeats composed of one amino acid (homorepeats) or a few amino acids (short tandem repeats - STRs). The proposed algorithm scans a sequence and builds a graph from a sequence *k*-mers and converts them into cycles. This step is presented in Figure S1.

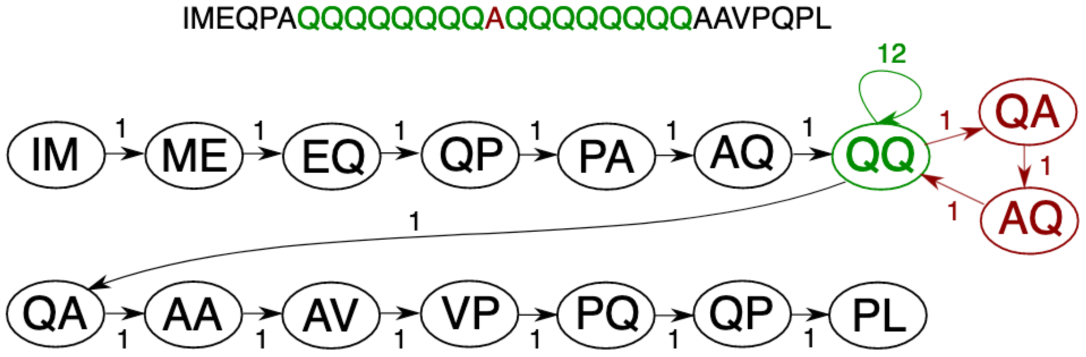

Figure S1. Graph-based searching for glutamine homorepeat with alanine insertion.The window is moved along the sequence creating nodes of a size *k*=2. A cycle (green) represents found homorepeat with alanine insertion (red).

Each graph node is created from *k*-mer of the sequence (here *k*=2). The positions of each related *k*-mers are tagged and transitions are connected to neighbouring *k*-mers. Cycles found in the graph represent repeats in the sequence and each cycle which occurs more often than a given threshold represents a repetitive region. The method is designed in such a way that it also finds imperfect tandem repeats (repeats with insertions in between repeats and mutations of amino acids within the repetitive region) and fused repeats (repeats composed of more than one type of repeats).
