# Supplementary material for: PlaToLoCo: the first web meta-server for visualization and annotation of low complexity regions in proteins
Source: Nucleic Acids Res. 2020 May 18;48(W1):W77–84. doi: 10.1093/nar/gkaa339 (PMC7319588; doi:10.1093/nar/gkaa339)
Supplement: gkaa339_Supplemental_Files [file gkaa339_supplemental_files.zip › Suppl2.pdf]

SUPPLEMENTARY MATERIAL 2

To investigate how each of the methods behaves on different datasets and to compare the runtime of selected LCR detection methods (a means of computational performance; Table S1) and the quantity of detected LCRs (Table S2), we ran all the methods on sequences from different proteomes and we used PDB as a control. Proteomes from different organisms were obtained from UniPort (March 2019, with cd-hit redundancy filtering in 4 steps: 90%, 70%, 50%, 40%) and we used the non-redundant PDB chain set the PDB database (https://www.ncbi.nlm.nih.gov/Structure/VAST/nrpdb.html, version from March, 2019, p=10e-7).

Table S1. Running time measured on different proteomes and on PDB.

Proteome	Sequences	Residues	Running time (min)
ARATH *Arabidopsis thaliana* (Mouse-ear cress)	15 884	6 505 776	62
BACSU *Bacillus subtilis* (strain 168)	3 886	1 113 428	20
CAEEL *Caenorhabditis elegans*	15 360	6 711 294	64
DANRE *Danio rerio* (Zebrafish) (*Brachydanio rerio*)	14 704	8 738 054	51
DROME *Drosophila melanogaster* (Fruit fly)	11 954	6 742 168	57
ECOLI *Escherichia coli* (strain K12)	3 953	1 230 745	19
HUMAN *Homo sapiens* (Human)	14 238	8 475 786	73
METJA *Methanocaldococcus jannaschii* (strain ATCC 43067 / DSM 2661 / JAL-1 / JCM 10045 / NBRC 100440) (*Methanococcus jannaschii*)	1 694	483 423	8
PLAF7 *Plasmodium falciparum* (isolate 3D7)	4 940	3 896 130	31
YEAST *Saccharomyces cerevisiae* (strain ATCC 204508 / S288c) (Baker's yeast)	5 270	2 548 147	20
PDB	14 461	483 423	30
**Total:**	**106 344**	**49 318 838**	**415**

Quantitative results produced by each method are shown in Table S2. From this table we can read that BACSU proteome contains irregular LCRs (i.e. LCRs that do not contain clear repeat patterns) found mostly by methods based on entropy-like statistics (e.g. SEG). On the other hand the METJA proteome contains LCRs that are more regular and were mostly found by methods specialized for repeat identification (e.g. GBSC).

Table S2. Total number of residues found by each method.

Proteome	Number of residues in LCRs				
	**SEG**	**CAST**	**fLPS**	**SIMPLE**	**GBSC**
ARATH *Arabidopsis thaliana* (Mouse-ear cress)	498 602	1 075 544	4 695 890	52 533	37 899
BACSU *Bacillus subtilis* (strain 168)	44 288	45 568	668 508	1 008	879
CAEEL *Caenorhabditis elegans*	521 656	1093 499	5 081 205	39 647	38 439
DANRE *Danio rerio* (Zebrafish) (*Brachydanio rerio*)	732 210	1 995 121	7 138 291	43 312	53 631
DROME *Drosophila melanogaster* (Fruit fly)	784 188	1 770 045	5 427 868	11 0678	93 182
ECOLI *Escherichia coli* (strain K12)	50 921	39 277	737 010	935	1 125
HUMAN *Homo sapiens* (Human)	810 998	1 834 901	7 151 468	47 616	56 295
METJA *Methanocaldococcus jannaschii* (strain ATCC 43067 / DSM 2661 / JAL-1 / JCM 10045 / NBRC 100440) (*Methanococcus jannaschii*)	28 406	17 065	436 828	263	370
PLAF7 *Plasmodium falciparum* (isolate 3D7)	759 283	2 070 029	3 843 999	53 958	153 613
YEAST *Saccharomyces cerevisiae* (strain ATCC 204508 / S288c) (Baker's yeast)	176 012	469 362	2 006 227	11 229	12 916
PDB	104 208	82 647	1 464 298	15 509	11 240
**Total:**	**4 510 772**	**10 493 058**	**38 651 592**	**376 688**	**459 589**

Overlaps between all found residues for each method can reveal the level of similarity among them. Table S3 presents the overlap of number of residues as well as the percentage agreement in between the methods. It is clear that results can significantly vary among methods. Such results clearly show that there is a need to provide the scientific community with the set of easily accessible tools for detection of LCRs as all these methods are designed to find different types of LCRs.

Table S3. Overlap between methods (number of detected low complexity residues and percentage in all datasets)

**Overlap (residues)**	**CAST**	**fLPS**	**SIMPLE**	**GBSC**
**SEG**	2 879 926 (5.84%)	4 331 357 (8.78%)	318 373 (0.65%)	436 202 (0.88%)
**CAST**		10 305 978 (20.9%)	299 459 (0.61%)	421 782 (0.86%)
**fLPS**			366 716 (0.74%)	457 825 (0.93%)
**SIMPLE**				175 682 (0.36%)
